# Kainate Receptors in Sensory Neurons: Molecular Identity and Functional Relevance to Pain

**DOI:** 10.1007/s12264-026-01594-6

**Published:** 2026-02-20

**Authors:** Sofía Degiorgi, Juan Lerma

**Affiliations:** Instituto de Neurociencias, UMH-CSIC, 03550 San Juan de Alicante, Spain

**Keywords:** Kainate receptors, Pain, Nociception, Transcriptomics, Dorsal Root Ganglion, GRIK1–5

## Abstract

Kainate receptors (KARs) are members of the ionotropic glutamate receptor family and possess diverse structural and functional properties that play critical roles in synaptic signaling, plasticity, and neural development. Several KAR subunits are strongly expressed in dorsal root ganglion neurons, and while their involvement in pain has been suggested, their precise role remains unclear. This review re-evaluates the roles of KARs in sensory physiology, with a particular focus on pain mechanisms. By combining recent single-cell transcriptomic data from dorsal root ganglia neurons with experimental evidence on KAR diversity, signaling, and function, we highlight how these receptors may shape sensory processing under normal and pathological conditions.

## Introduction

Kainate receptors (KARs) are members of the ionotropic glutamate receptor family, alongside N-methyl-D-aspartate (NMDA) receptors and α-amino-3-hydroxy-5-methyl-4-isoxazole propionic acid (AMPA) receptors. It is now well established that these receptors mediate fast excitatory neurotransmission in the central nervous system.

**Fig. 1:**
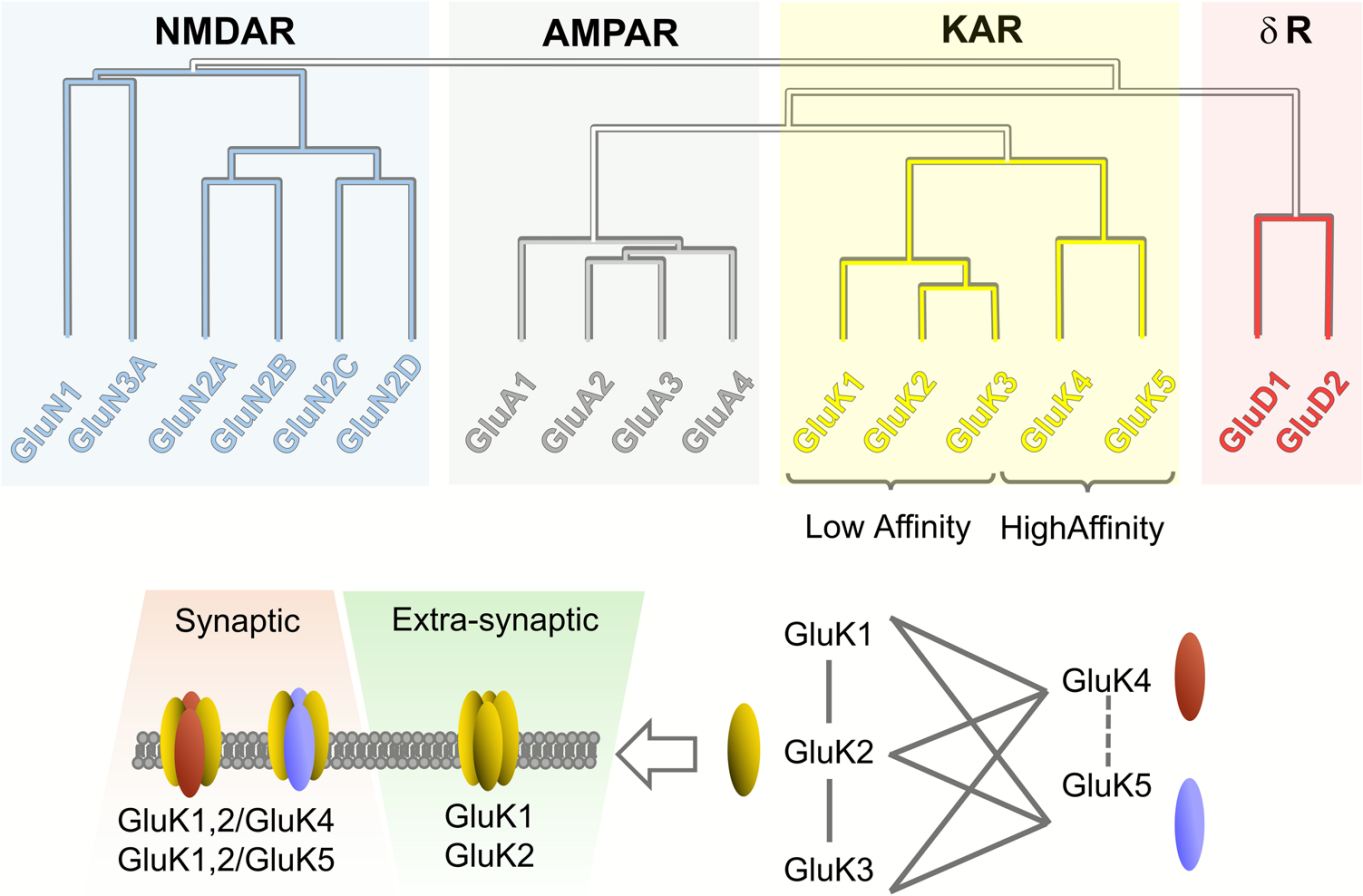
Ionotropic glutamate receptor families grouped by sequence homology. NMDA receptors (NMDAR) are assembled from combinations of six subunits, whereas AMPA receptors (AMPAR) are composed of four closely related subunits. Kainate receptors (KAR) comprise two subfamilies: low-affinity (GluK1–3) and high-affinity (GluK4–5) subunits. GluK1–3 can form functional homomeric or heteromeric receptors, while GluK4 and GluK5 must co-assemble with GluK1–3 to yield functional channels. Importantly, GluK4/5 are required for synaptic targeting of KARs; otherwise, receptors remain extrasynaptic [3]. Delta glutamate receptors (δ R) consist of two subunits that, unlike other ionotropic glutamate receptors, do not gate ions in response to glutamate. Instead, they act as synaptic organizers and scaffolding proteins, contributing to synapse formation and plasticity.

Structurally, KARs are tetrameric assemblies composed of five subunits, which are grouped into two subfamilies based on structural and pharmacological distinctions. GluK1, GluK2, and GluK3 exhibit low affinity for kainate, whereas GluK4 and GluK5 are considered high-affinity subunits (Fig. 1). GluK4 and GluK5 share approximately 68% sequence identity with each other but only around 42% with GluK1–3, whereas GluK1–3 show 75%–80% identity among them [1]. Most of the variability among these subunits lies in their amino- and carboxyl-terminal domains. The genes encoding these subunits are designated *GRIK1* to *GRIK5*. GluK1–3 can form either homomeric or heteromeric channels. In contrast, the high-affinity subunits GluK4 and GluK5 only produce functional receptors when co-assembled with GluK1–3 subunits (see [2] for details). However, GluK4 and GluK5 subunits are fundamental for targeting KARs to the synapse [3]. Furthermore, studies have shown that KARs play a critical role in modulating synaptic transmission and may contribute to the regulation of excitatory and inhibitory signaling in the brain [4, 5]. KARs are involved not only in baseline excitatory transmission, but also in certain forms of plasticity such as long-term potentiation (LTP) and depression (LTD), which are fundamental for learning and memory [5–8].

KARs share the conserved tetrameric architecture of other ionotropic glutamate receptors, with each subunit comprising an extracellular N-terminal domain, three transmembrane segments (M1, M3, M4), a pore-forming M2 loop, and a cytoplasmic C-terminal tail [2, 9]. The ligand-binding domain (LBD) is extracellular, formed by discontinuous S1 and S2 segments, adopts a bilobed structure whose interdomain contacts shape ligand affinity, deactivation, and desensitization kinetics, distinguishing KARs from AMPA receptors [10–12]. The M2 region, rich in hydrophobic residues, forms a hairpin-like structure that determines ion selectivity. The arrangement of amino acids that forms the pore confers to KARs its characteristic Na^+^ and K^+^ permeability, and certain KARs are also permeable to Ca^2+^ and Cl^-^ [13]. In addition, crystallographic studies have revealed the existence of an ion-binding pocket in KAR subunits, underlying a sodium-dependent gating mechanism absent in AMPA receptors [14–16]. Removal of Na⁺ accelerates desensitization in GluK2-containing receptors, an effect independent from membrane potential and related to cation size, which is linked to residue M770 in the S2 domain [14].

The robust expression of KARs in DRG neurons and dorsal horn circuits implicates them as key modulators of nociceptive processing. Early pharmacological studies in immature rodents demonstrated that application of kainate induces strong depolarizing currents in DRG neurons, and that C-fiber evoked potentials in the dorsal roots are significantly diminished or abolished by kainate exposure [17]. Complementary electrophysiological investigations using patch clamp recordings revealed that a subpopulation of dissociated DRG neurons, classified as C-fibers, display desensitizing inward currents in response to kainate and other agonists such as domoate and quisqualate [18], suggesting that KARs are strategically positioned to influence nociceptive signaling. The development of pharmacological agonists and antagonists, along with the generation of advanced knockout mouse models, has significantly contributed to understanding the physiology of KARs but has not resolved their contributions to distinct sensory modalities, their precise synaptic roles in DRG neurons and spinal circuits, as well as the mechanisms by which KARs may modulate pain states *in vivo*.

In this review, we undertake a comprehensive re-examination of the potential roles of KARs in sensory physiology, with particular emphasis on their contribution to pain mechanisms. This analysis is motivated by the availability of recent transcriptomic datasets from DRG neurons, which provide an unprecedented resolution of KAR subunit expression patterns. We further integrate these findings with the extensive experimental evidence that has progressively delineated the molecular diversity, signaling properties, and physiological functions of KARs. Together, these advances allow for a more refined understanding of how KARs may participate in shaping sensory processing under both normal and pathological conditions.

## Expression of KARs in Dorsal Root Ganglion Cells. Transcriptomic Harmonized Analysis

Peripheral sensory neurons have historically been classified based on an integrated set of morphological, physiological, and functional characteristics, including size, degree of myelination, conduction velocity, and the nature of the stimuli they transduce. Large-diameter, heavily myelinated A-fibers conduct rapidly (2–55 m/s) and are primarily involved in the detection of non-noxious mechanical stimuli. In contrast, small-diameter, unmyelinated C-fibers conduct slowly (<2 m/s) and respond to a broader range of modalities, including thermal, mechanical, and chemical stimuli [19, 20].

According to their mechanical sensitivity thresholds, neurons that respond robustly to innocuous mechanical stimuli applied to the skin are termed low-threshold mechanoreceptors (LTMRs) and include Aβ-, Aδ-, and C-fiber subtypes, corresponding to fast, intermediate, and slow conduction velocities, respectively. Among Aβ LTMRs, further subdivision is based on adaptation dynamics to sustained skin indentation, distinguishing rapidly adapting mechanoreceptors (RAMs) from slowly adapting mechanoreceptors (SAMs) [21]. High-threshold mechanoreceptors (HTMRs) require stronger stimuli and may conduct via Aδ- or C-fibers, while polymodal nociceptors—mechano-heat (MH), mechano-cold (MC), and mechano-heat-cold (MHC)—respond to multiple modalities [21, 22]. Additionally, some C-fiber neurons are selectively responsive to noxious heat (C-Heat) or cold (C-Cold), lacking mechanical sensitivity [20]. A further subclass of C-fiber neurons, termed ‘silent’ or ‘sleeping’ nociceptors, are initially unresponsive but become mechanosensitive or thermosensitive following inflammation, a process known as sensitization, a hallmark of chronic pain that lowers activation thresholds and enhances excitability [21].

Molecular markers have refined this framework, identifying transcriptionally distinct populations: IB4-positive non-peptidergic (NP) neurons, peptidergic (PEP) neurons expressing CGRP and substance P, and tyrosine hydroxylase (Th)- positive subtype. Larger-diameter neurons typically express neurofilament 200 (NF200), a marker of myelinated fibers [23]. Together, these functional and molecular classifications define the complex heterogeneity of somatosensory neurons.

However, classification systems based solely on diameter, axonal properties, or limited molecular marker panels are biased and lack the resolution to fully capture DRG neuron heterogeneity, thereby limiting our understanding of their roles in modalities such as touch, itch, pain, temperature perception, and proprioception. Recent advances in single-cell and single-nucleus RNA sequencing (sc-RNA-seq and snRNA-seq, respectively) have overcome these limitations, providing unbiased, high-resolution classification of DRG neurons based on their transcriptional profiles. These approaches have uncovered discrete neuronal populations, identified conserved and species-specific features, and enhanced translational relevance from rodent models to primates and humans [20, 23–38].

This development has also introduced considerable variability in how these subtypes are annotated across studies. Technical differences, such as tissue preparation methods, sequencing depth, the number of cells or nuclei analyzed, and the computational parameters used for clustering and dimensionality reduction, can lead to differences in the number, identity, and nomenclature of neuron types identified in each dataset. These inconsistencies have made it difficult to directly compare neuronal populations across studies and species, highlighting the need for a standardized framework. To overcome this, Bhuiyan *et al.* (2024) [25] recently developed comprehensive harmonized reference atlases using sc/snRNA-seq data from 23 DRG datasets across six mammalian species. These atlases define 18 neuronal and 11 non-neuronal cell types using standardized nomenclature, enabling robust cross-study and cross-species comparisons. This framework enhances the resolution of rare and lowly expressed cell types and integrates previously fragmented datasets to create a unified view of sensory neuron diversity.

**Fig. 2:**
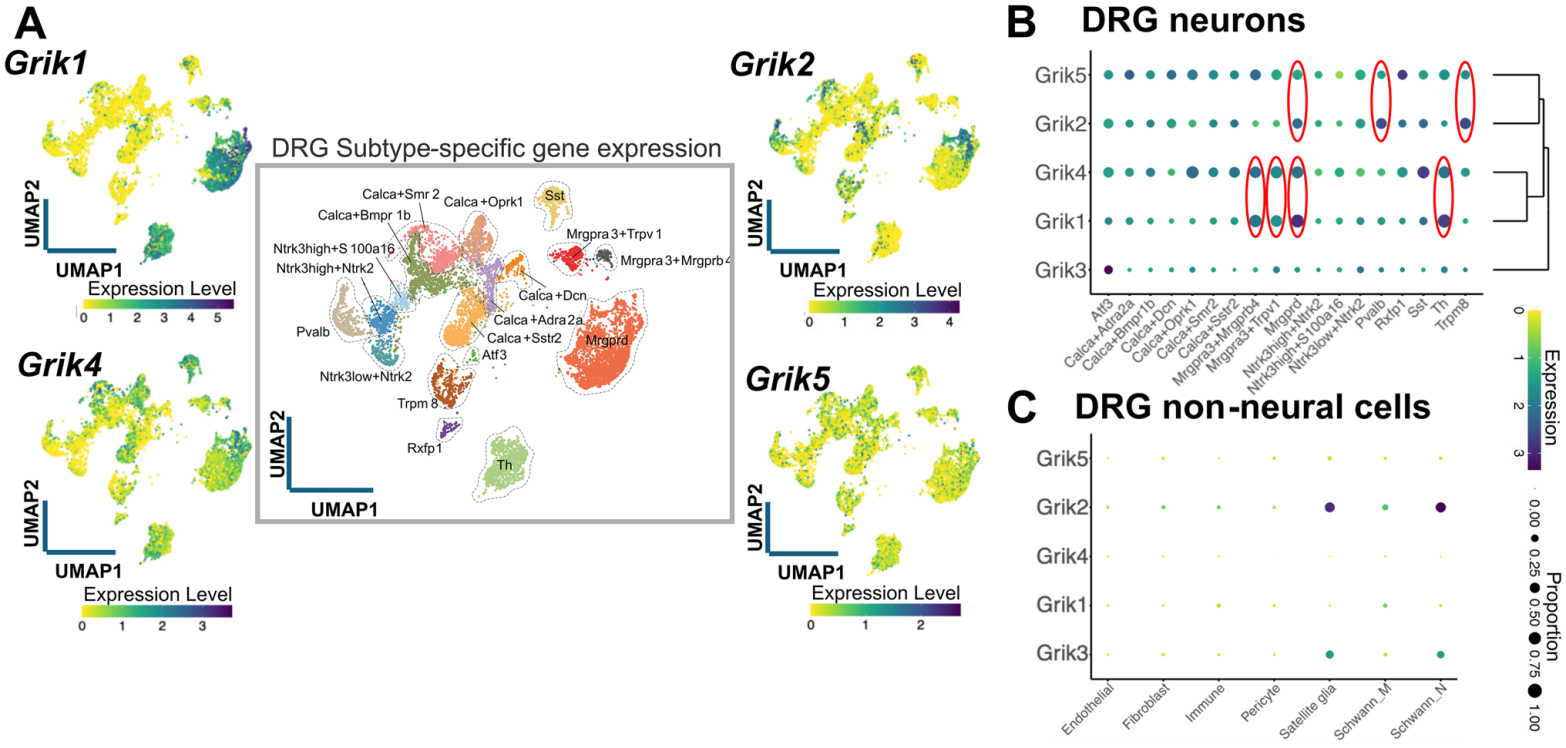
Subtype-specific gene expression in DRG neurons and non-neural cells. **A** Each dot on the UMAP projection represents a cell or nucleus from one of 21 integrated datasets (central plot), where the harmonized DRG neuronal reference atlas contains 18 neuronal subtypes across 75,928 cells/nuclei. Of these subtypes, specific functions have been ascribed to 15 of them, and identified as A-fiber subtypes proprioceptors (Pvalb); Aβ-rapid-adapting–low-threshold mechanoreceptors (Ntrk3 high+Ntrk2); Aβ-field/slow-adapting–low-threshold mechanoreceptors (Ntrk3 high+S100a16); Aδ-low-threshold mechanoreceptors (Ntrk3 low+Ntrk2); Aδ–high-threshold mechanoreceptors (Calca+Bmpr1b and Calca+Smr2); C-fiber subtypes nociceptors (Calca+Sstr2); nociceptors (Calca+Adra2a and Mrgprd); cold thermoreceptors (Trpm8); C-low-threshold mechanoreceptors (Mrgpra3+Mrgprb4); pruriceptors (Mrgpra3+Trpv1 and Sst) as well as Atf3 cluster expressing injury-induced transcription factors such as Atf3, Sox11, and Jun. Levels of expression of each KAR subunit are shown as specific UMAP plots, where the intensity of expression in each cell is color-coded (the yellow colour indicates each cell with no expression). Note the different scales for each expression map. **B** Heatmap of kainate receptor gene expression in DRG neurons grouped by cell type. Colour intensity reflects average expression levels, while dot size represents the proportion of expressing cells. Two distinct cell clusters emerge according to the expression of KARs: one formed by *Grik1–Grik4* and the other by *Grik2–Grik5*. Neuronal subtypes showing preferential and complementary expression of each subunit pair are indicated. **C** Gene expression heatmap for non-neural DRG cells. *Grik2* is the most abundantly expressed gene in two specific glial cell types, satellite glia and non-myelinating Schwann cells (Schwann_N), along with *Grik3*. Data elaborated from the Harmonized DRG and TG reference atlas website (https://painseq.shinyapps.io/harmonized_painseq_v1/) (see [25]).

Among the 18 neuronal populations identified, 15 correspond to subtypes with previously characterized physiological roles in rodent models where genetic access was enabled through Cre-driver lines. These include several A-fiber subtypes such as Pvalb-expressing proprioceptors; Ntrk3^high^+Ntrk2 Aβ-rapid-adapting (RA) LTMRs; Ntrk3^high^+S100a16 Aβ-field/slow-adapting (SA) LTMRs; Ntrk3^low^+Ntrk2 Aδ-LTMRs; Calca+Bmpr1b Aδ-HTMRs; and Calca+Smr2 Aδ-HTMRs. In addition, the atlas includes multiple C-fiber subtypes such as Calca+Sstr2 nociceptors; Calca+Adra2a nociceptors; Trpm8 cold thermoreceptors; Th-expressing C-LTMRs; Mrgprd nociceptors; Mrgpra3+Mrgprb4 C-LTMRs; Mrgpra3+Trpv1 pruriceptors; and Sst pruriceptors. One further cluster expresses the injury-induced transcription factors Atf3, Sox11, and Jun, and is likely associated with nerve injury or stress responses. In addition to these functionally annotated types, three transcriptomically distinct C-fiber subtypes without clearly defined physiological functions were identified. These include a Calca+Dcn population co-expressing Ntrk2; a Calca+Oprk1 cluster that also expresses Npy1r; and a rare Rxfp1 subtype distinguished by its exceptionally high expression of *Trpv1*. To address the limitations of functional annotation, the authors propose a dual strategy in which neuronal subtypes are classified primarily based on the expression of key molecular markers. To further support cross-study comparisons of transcriptomically defined cell types, regardless of nomenclature, they designed the harmonized atlas metadata to enable rapid name conversion between all datasets (Fig. 2).

Within the described neuronal populations, KAR subunits are expressed in a heterogeneous manner across different cell types. Notably, *Grik1* and *Grik4* are expressed in over half of DRG neurons (52.4% and 63%, respectively), whereas *Grik2*, *Grik3*, and *Grik5* show lower expression levels (40%, 12%, and 48%, respectively). Interestingly, among the KAR subunits, *Grik1* is the most enriched, showing a sevenfold higher expression in IB4⁺ cells. In contrast, *Grik2*, *Grik3*, and *Grik5* do not display any selective expression between IB4⁺ and IB4⁻ populations [39]. *Grik4* expression is also significantly elevated in IB4⁺ cells, although to a lesser extent than *Grik1.* This distribution suggests, however, a preferential expression of certain KAR subunits in DRG neurons (Fig. 2A), underscoring the potential functional relevance of KARs in sensory physiology.

Among DRG subpopulations, *Grik1* expression is most prominent in the Mrgprd and Th neuronal subtypes, where it is detected in 93.2% of approximately 2,600 Mrgprd neurons and in 96.3% of roughly 1,100 Th neurons examined. High levels of *Grik1* expression are also observed in Mrgpra3+Mrgprb4 (84.0% of 119 neurons) and Mrgpra3+Trpv1 (74.0% of 311 neurons) populations, while lower, yet notable, expression is detected in Sst neurons, where it is present in 33.1% of about 600 cells. In contrast, *Grik2* expression is enriched in Trpm8 neurons, where it is detected in 69.3% (352 cells), and in the Pvalb population (62.8 %; 289 neurons). It is also significantly expressed in Mrgprd (56.2% of 2,627 neurons), Atf3 (51.6%), and Sst (42.2% of 619 neurons) populations, suggesting a broader, though less specific, distribution across multiple subtypes. *Grik3* is poorly expressed in DRG neurons, being detected only in 12% of DRG neurons. Unlike *Grik3*, which shows no clear enrichment in any specific subtype, *Grik4* exhibits a broader expression profile, being detected at relatively high levels across multiple DRG populations. Notably, it is expressed in 81.6% of Th neurons, 81.5% of Calca+Oprk1 (393 neurons), 80.1% of Mrgpra3+Trpv1 (249 neurons), 79.0% of Mrgprd (2076 neurons), 79.0% of Sst (489 neurons), 73.95% of Mrgpra3+Mrgprb4 (88 neurons), and 63.3% of Calca+Sstr2 (654) expressing cells. This widespread distribution suggests that *Grik4* is not restricted to a specific neuronal subtype but rather broadly expressed among nociceptive and low-threshold mechanosensory neurons. Similarly, *Grik5* does not exhibit subtype-specific enrichment, but instead shows low to moderate expression across most sensory neuron populations, typically present in approximately 50% of the cells within each cluster. Furthermore, the substantial proportion of non-overlapping expression between *Grik1* and *Grik2*, with only half of the neurons expressing *Grik1* also expressing *Grik2*, suggests that KAR subunits may also operate autonomously, supporting distinct and potentially non-redundant roles in glutamatergic signaling within sensory ganglia (Fig. 2B). Altogether, *Grik1* emerges as the most widely coexpressed subunit, showing strong overlap with *Grik4* (nearly 80% of all *Grik1* neurons express *Grik4*) and substantial coexpression with *Grik5* (59%). Given that high-affinity KAR subunits cannot form functional receptors on their own and must instead associate with low-affinity subunits (GluK1–GluK3) to generate heteromeric KARs, a broad coexpression of both subunit types would be expected within the same neuronal populations. Transcriptomic data indicate that *Grik4* is the most commonly expressed high-affinity subunit, present in 75%–82% of cells that express at least one low-affinity subunit. As anticipated, *Grik4* and *Grik5* show partial coexpression too (*Grik4* is expressed in nearly 78% of *Grik5*-expressing cells). These data suggest that GluK4 is the preferred high-affinity subunit for the assembly of heteromeric KARs in sensory neurons, serving as a central hub in KAR assembly within DRG populations. Overall, the analysis reveals two distinct clusters: one composed of GluK1 and GluK4, predominantly expressed in Mrgpra3+Mrgprb4, Mrgpra3+Trpv1, Mrgprd, and Th cells; and another comprising GluK2 and GluK5, mainly present in Mrgprd, Pvalb, and Trpm8 cells (Fig. 2B).

On the other hand, single-cell transcriptomic profiling reveals that KAR subunit expression is not limited to neurons. *Grik2* is highly prevalent in satellite glia (88.54% of cells) and non-myelinating Schwann cells (90.22%), with moderate levels in myelinating Schwann cells (28.14%) and minimal expression in endothelial cells, fibroblasts, immune cells, and pericytes (Fig. 2C). Although the functional significance of kainate receptor expression in peripheral glial populations is largely unexplored, the high levels of *Grik2* in satellite glial cells and non-myelinating Schwann cells suggest that KARs may contribute to several key aspects of glial physiology. Satellite glial cells tightly envelop sensory neuron somata and regulate extracellular ion composition, neurotransmitter clearance, and neuron–glia signaling [40, 41]. In this perisomatic microenvironment, ionotropic or metabotropic signaling through GluK2-containing receptors could modulate K⁺ buffering, regulate glial membrane potential, or influence Ca^2^⁺-dependent pathways that shape neuron–glia interactions. These mechanisms are particularly relevant because satellite glial cells undergo profound phenotypic changes during inflammation, neuropathic pain, and nerve injury, including increased coupling, altered excitability, and enhanced responsiveness to glutamate [42, 43].

Similarly, elevated *Grik2* expression in Schwann cells raises the possibility that KARs participate in axonal support, myelin maintenance, or injury-induced remodeling [44]. Schwann cells express multiple glutamate receptors and exhibit glutamate-evoked Ca^2^⁺ signals that modulate proliferation, cytokine release, and myelin protein expression. GluK2, which forms functional homomeric receptors and does not require auxiliary subunits for surface expression, could provide a direct route for Schwann cells to sense glutamatergic signals arising from sensory axons or from injury-associated extracellular glutamate elevations.

Taken together, the high expression of *Grik2* in peripheral glial cells, combined with their strategic anatomical positions and well-established roles in metabolic support, immune signaling, and chronic pain, suggests that KAR signaling within these glial populations could influence both normal sensory processing and pathological pain states. Although experimental evidence is still lacking, these transcriptomic data highlight an important area for future investigation.

## Molecular, Signaling, and Functional Features of KARs

### Structural Diversity of KARs

An important aspect of KARs is that, as in other ionotropic receptors, they achieve considerable molecular diversity beyond their five canonical subunits (GluK1–GluK5) through RNA editing and alternative splicing, which enhances their functional complexity. One of the best-characterized modifications is RNA editing at the Q/R site within the M2 domain of the GluK1 and GluK2 subunits (but not GluK3, GluK4, and GluK5) [45, 46]. The substitution of glutamine (Q) with arginine (R) reduces calcium permeability [47], alters current–voltage relationships (shifting from inwardly rectifying to linear or slightly outward), and significantly lowers single-channel conductance [48]. GluK2 also undergoes editing at two additional sites (I/V and Y/C) in the M1 domain [49], further diversifying its functional properties. In contrast, receptors incorporating the unedited GluK1(Q) or GluK2(Q) subunits display some calcium permeability [10, 47], and approximately 25-fold larger single-channel conductance compared to their edited counterparts [48]. Additionally, they exhibit strong inward rectification due to voltage-dependent channel block by intracellular polyamines [50]. Importantly, edited and unedited subunits can co-assemble, forming heteromeric receptors with intermediate electrophysiological properties [51].

Alternative splicing also contributes substantially to structural and functional variation, particularly in GluK1, GluK2, and GluK3, mainly affecting their cytoplasmic C-terminal domains [52–55]. In contrast, no splice variants have been reported for GluK4 or GluK5 subunits. GluK1 isoforms include GluK1-1/2 (differing in an N-terminal insert) [56] and C-terminal variants such as GluK1-2a, -2b, -2c, and the human-specific GluK2d [52, 53, 57], affecting intracellular trafficking, receptor localization, and protein-protein interactions [10]. GluK2 exists as GluK2a and GluK2b isoforms, which co-assemble and share electrophysiological profiles but differ in C-terminal tail structure [1, 58]. GluK3 isoforms (GluK3a/b) diverge at their C-terminal ends [54] and differ in glutamate affinity and trafficking efficiency, with GluK3a exhibiting higher surface expression due to a forward trafficking motif that also exists in GluK2a, promoting higher plasma membrane expression compared to GluK3b [59, 60].

Collectively, these editing and splicing events generate a rich repertoire of structurally and functionally distinct KARs, far exceeding the diversity predicted by their primary gene count, enabling precise tuning of KARs' roles in synaptic transmission and plasticity in different neural circuits.

### Desensitization of KARs

A defining feature of KARs is their rapid and pronounced desensitization in response to sustained agonist exposure, with kinetics determined by subunit composition and agonist type [2, 61]. Desensitization occurs most rapidly in homomeric GluK2 receptors (τ ∼ 1.5 ms), is markedly slowed by the incorporation of GluK5 subunits, and reaches its slowest kinetics in homomeric GluK3 receptors (∼8 ms) [54, 62].

Recovery from desensitization is markedly slower and is influenced by the nature of the agonist, ranging from ~15 s after glutamate to ~1 min after kainate [63]. In general, the time course of recovery of KARs is significantly slower than that of AMPA receptors. Subunit composition also plays a pivotal role: while homomeric GluK1 receptors exhibit a biphasic recovery (with fast and slow components at ~50 ms and ~5 s, respectively), heteromeric GluK1/GluK5 receptors recover more uniformly and rapidly (~12 s) [2, 62].

The desensitization and recovery dynamics in KARs indicate that the equilibrium between the desensitized and active states is highly biased toward the desensitized form, especially under sustained activation. This suggests that KARs spend most of their time in the desensitized state after activation and that a large fraction of channels is already inactive before they can be activated [2, 8]. Moreover, the overlap between the activation and desensitization curves of KARs implies that certain agonist concentrations can simultaneously promote channel opening and partial desensitization. This suggests that a significant proportion of KARs may already be inactivated at rest, even before synaptic stimulation, which has important physiological implications. However, Neto1 and Neto2 (neuropilin- and tolloid-like proteins 1 and 2) act as auxiliary subunits of KARs. They critically regulate receptor properties, including gating, trafficking, and synaptic localization (e.g., see [64], thereby shaping the contribution of KARs to excitatory transmission and plasticity. Both proteins slow the deactivation and desensitization of KAR currents, thus modulating KAR-mediated synaptic responses [3]. In the peripheral nervous system, Neto2 is expressed in a small subset of DRG neurons, ranging from 1%–50% across different populations [25]. Their presence could influence KAR desensitization properties in sensory neurons, although their impact is likely less pronounced than that described in central neurons.

### Pharmacological Profile

A longstanding obstacle in the study of KARs has been the limited availability of highly selective pharmacological tools. While there is a well-established pharmacological distinction between NMDA receptors and other ionotropic glutamate receptors, the situation is more complicated for AMPA and KARs, which share several common agonists and antagonists. This overlap in pharmacological profiles has hindered the study of KARs for years [2, 65]. Progress in the development of new pharmacological agents for studying KARs has been relatively limited, and many of the compounds currently in use are still based on discoveries made decades ago.

Initially, both AMPA and KARs were often grouped together as "non-NMDA" receptors, complicating the understanding of their individual roles in synaptic transmission. The identification of distinct subunits for both AMPA and KARs and the development of selective antagonists, however, have clarified this classification. Notably, the discovery of the AMPA-specific antagonist GYKI 53655 (also known as LY300168), which does not affect KARs, has significantly advanced the functional characterization of KARs [66]. The development of other subunit-specific drugs and the use of genetically modified mice lacking KAR subunits have been pivotal in advancing our knowledge of KARs in synaptic physiology [5].

#### *Agonists*

 Early studies on KAR pharmacology relied on naturally occurring agonists such as kainic acid and domoic acid. While these compounds helped establish the basic functional profile of KARs, their limited selectivity over AMPA receptors complicated the interpretation of experimental data. Domoic acid, for instance, is approximately 20–25 times more potent than kainate in DRG neurons and recombinant GluK1-containing receptors [18, 52] yet still exhibits activity at GluK3 subunits and displays a slower desensitization profile, reducing its suitability as a selective pharmacological tool [54]. The subsequent introduction of GYKI 53655 provided an effective strategy to pharmacologically isolate KAR-mediated responses by selectively antagonizing AMPA receptors, without affecting KARs [66]. Significant progress was made with the synthesis of ATPA (2-Amino-3-(3-hydroxy-5-tert-butylisoxazol-4-yl) propanoic acid), a substituted analogue of AMPA that displays marked selectivity for GluK1-containing KARs. ATPA has an EC_50_ of 1 μmol/L for GluK1-containing receptors but also activates heteromeric KARs composed of GluK2 and GluK5, albeit with lower affinity (EC_50_ ≈ 20 μmol/L) [67]. ATPA is significantly more effective than kainate, with an EC_50_ of 0.6 μmol/L for native KARs in DRG neurons and 2.1 μmol/L for recombinant GluK1 subunits [68], while its affinity for AMPA receptors is much lower—about 500 times less potent (EC₅₀ ≈ 340 μmol/L in cerebellar Purkinje cells). This high potency and selectivity profile makes ATPA an invaluable pharmacological tool for dissecting the physiological and pathological roles of GluK1-containing KARs. Other notable KAR agonists include (S)-5-iodowillardiine [62, 68, 69] and SYM 2081, the latter showing high selectivity for KARs over AMPA receptors and inducing fast-desensitizing currents, though with less defined subunit specificity than ATPA; due to its rapid desensitization at low concentrations, SYM 2081 can also act as a functional antagonist [70, 71]. Collectively, however, the pharmacological study of KARs has been challenging due to the overlap of agonists and antagonists shared with AMPA receptors. Perhaps the discovery of agonists such as ATPA and the use of receptor subtype-specific antagonists (see below) have enabled more accurate delineation of the role of KARs in synaptic transmission, plasticity, and excitotoxicity.

#### *Antagonists*

 The ability to pharmacologically separate AMPA and KAR function has been substantially improved by the identification of ligands with high subtype specificity. Early non-NMDA antagonists such as CNQX and NBQX were useful in blocking both receptor types, but their limited selectivity restricted their value for dissecting KAR-specific contributions. A breakthrough in isolating KARs' activity came with the development of 2,3-benzodiazepines, particularly GYKI53655 and its stereoisomer LY303070. These compounds act as highly selective, noncompetitive antagonists of AMPA receptors, with negligible effects on KARs. Their introduction made it possible to block AMPA receptor–mediated currents while preserving KARs signaling, thereby resolving many of the early ambiguities in non-NMDA receptor pharmacology. The use of GYKI compounds has been instrumental in defining the physiological and pathological contributions of KARs in both synaptic transmission and plasticity [66]. An additional key advancement came with LY382884, a 6-substituted decahydroisoquinoline that exhibits a strong preference for GluK1-containing receptors over other KAR subtypes and AMPA receptors. This high degree of selectivity allowed more precise characterization of GluK1 function in both native tissues and recombinant systems, making it a valuable tool for studying peripheral and central roles of KARs [8, 72]. Among the more recent GluK1-selective ligands, UBP310 has emerged as a potent and highly specific antagonist, effectively blocking both native and recombinant GluK1-containing KARs in rodents and humans [73]. This compound, along with its predecessors such as UBP302 [74] and the more refined ACET [75], has provided an unprecedented level of pharmacological resolution for targeting GluK1 subunits. The disproportionate abundance of GluK1-selective ligands compared to GluK2 or GluK3 likely reflects structural differences in their ligand-binding domains [76]. Lectins, such as concanavalin A (ConA), selectively bind N-glycosylated residues on KARs and AMPA receptors, reducing ligand-induced rapid desensitization and serving as valuable tools for probing gating mechanisms and receptor dynamics [61, 77]. Additionally, extracellular ion modulation also plays a key role in KAR function: extracellular Na⁺ and Cl⁻ act through an allosteric mechanism involving residue M770 in the S2 segment of GluK2 [14, 78]. Proton sensitivity further differentiates KAR subtypes, as most KARs are inhibited by acidic pH, with the exception of GluK2/GluK4 heteromers; this inhibition is voltage-independent and can be relieved by extracellular spermine [79].

### Non-Canonical Signaling of Kainate Receptors

**Fig. 3:**
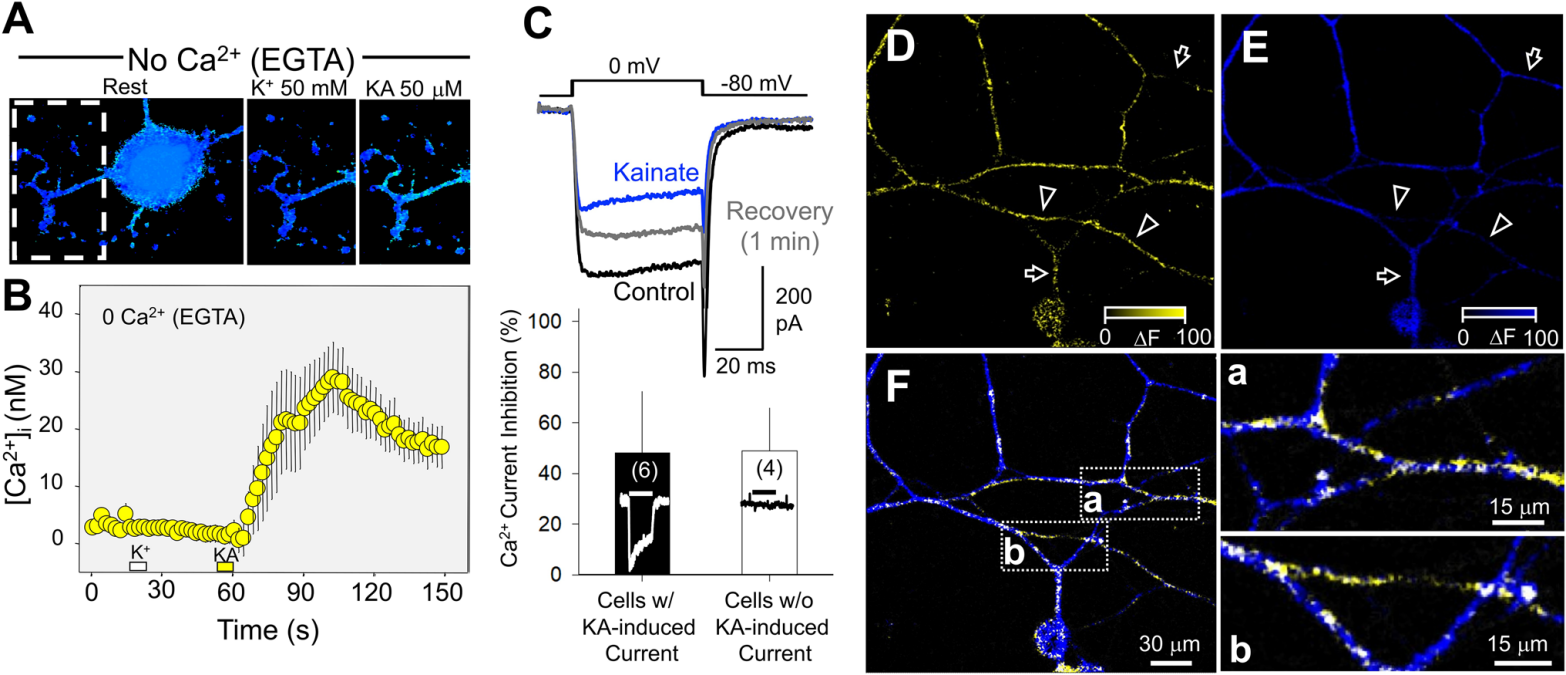
Properties of kainate receptors in DRG neurons. **A–B** Activation of kainate receptors triggers an increase in [Ca^2+^]_int_ through the release of Ca^2+^from intracellular stores. In the absence of extracellular Ca^2+^ (no added Ca^2+^ plus EGTA), kainate (KA), but not K^+^, is able to increase [Ca^2+^]_int,_ demonstrating the metabotropic action of KARs. Such an increase in [Ca^2+^]_int_, most likely results from the release of Ca^2+^ from intracellular stores induced by second messengers, was small (<=30 nmol/L) when compared to the overall increase induced by KA in the presence of extracellular Ca^2+^ (ca. 200 nmol/L, as a consequence of kainate-induced membrane depolarization) but consistently found in these cells. The images in **A** illustrate an example of KA-induced rise in [Ca^2+^]_int_ from intracellular stores in a neurite (box) from a dissociated DRG neuron. **B** shows the kainate-induced variation in [Ca^2+^]_int_ as an average from 18 neurons. Points are the mean+SEM. **C**. Kainate Receptor activation reversibly inhibits N-type voltage-dependent Ca^2+^ currents, irrespective of their ionotropic action. N-type currents were recorded under perforated patch whole-cell conditions and evoked by depolarizing pulses from −80 mV to 0 mV (top recordings). Similar levels of inhibition were observed in DRG cells either lacking (white bar) or expressing (black bar) ionotropic kainate receptors (examples of KA-induced currents are included within the bars), indicating that the permeation through the KAR ion channel was not required for non-canonical signalling. The inhibitory effect of KARs was abolished in cultures that had been treated with Pertussis toxin. Ionotropic and metabotropic KARs are unevenly distributed in dissociated DRG neurons and tend to be located at specific spots along neurites. **D–F** sites where KA-induced inhibition of Ca^2+^ currents took place were revealed by subtracting K^+^-induced peak Ca^2+^ confocal images before and after KA and coded in intensity of yellow colour (**D**; i.e., these correspond to KARs with metabotropic actions). Sites where KA induced Ca^2+^ responses (i.e., mostly induced by an ionotropic depolarizing action) were obtained and color-coded in blue (**E**). Arrowheads in (**D**) and (**E**) highlight points where inhibition was strong while the KA-induced Ca^2+^ increase was low; arrows point to areas where inhibition was negligible despite the fact that KAR were present as indicated by the induced robust Ca^2+^ signal. Both images are merged in **F**, where coincidences are coded in white colour. Details on the right better illustrate where metabotropic (yellow), ionotropic (blue), and overlapping activities (white) distribute along DRG neuron neurites. All data are taken from Rozas *et al*., 2003 ([80]). For more details, see this publication.

Although KARs are traditionally classified as ionotropic glutamate receptors mediating fast synaptic transmission via cation flux, they also exhibit metabotropic signaling capabilities. In no place has this function been more evident than in DRG neurons. In these cells, KAR activation can initiate G protein–dependent signaling cascades, independent of ion conductance [80] (Fig. 3). In other places of the nervous system, non-canonical mechanisms have been shown to modulate neuronal excitability and synaptic release of both glutamate [81] and GABA [82, 83] as well as long-term potentiation [84]. In DRGs, it might contribute to nociceptive signaling and pain modulation by regulating glutamate release of primary afferents onto lamina II dorsal horn neurons [80, 85]. In DRG neurons, exposure to kainate elicits a G protein–dependent signaling cascade that inhibits voltage-gated calcium channels (Fig. 3). Pharmacological studies further support the involvement of a pertussis-sensitive G-protein and protein kinase C in this cascade, as both pertussis toxin and PKC inhibitors (e.g., staurosporine, bisindolylmaleimide) abolish the kainate-induced inhibition. This signaling mechanism mirrors pathways activated by group I metabotropic glutamate receptors, involving phospholipase C and IP₃-mediated calcium release. Furthermore, the AMPA receptor antagonist LY303070 does not block these effects, confirming that they are specifically mediated by KARs. A similar noncanonical signaling cascade had been previously described in hippocampal inhibitory terminals [83], as well as in excitatory terminals [86] and postsynaptic sites [87]. Consistently, using confocal calcium imaging, Rozas *et al.* (2003) [80] demonstrated that kainate induced the release of Ca^2^⁺ from intracellular stores, probably induced by second messengers (Fig. 3A, B). Although modest in magnitude (around 30 nmol/L) compared to the overall increase induced by kainate in the presence of extracellular Ca^2+^ (~200 nmol/L), this release is consistent with the concept of metabotropic KARs.

**Fig. 4:**
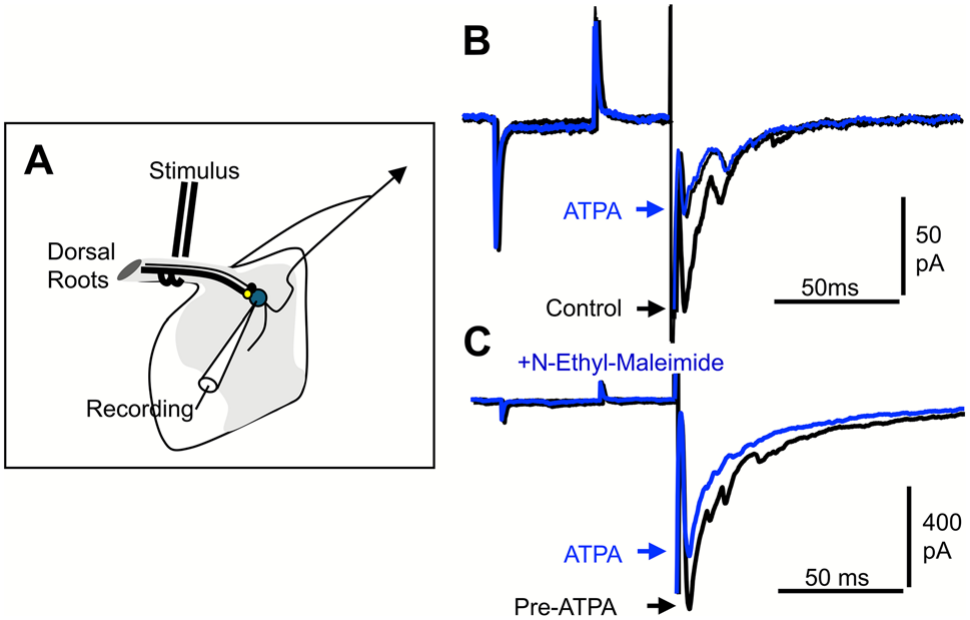
GluK1 KAR activation inhibits glutamate release from primary afferents involving the activation of a G protein. **A** Synaptic responses were recorded from dorsal horn neurons in spinal cord slices and evoked by dorsal root stimulation. Application of ATPA, the preferred agonist of GluK1-containing KARs, reversibly reduced the AMPAR-mediated EPSC (**B**). After treating slices with N-ethyl-maleimide to selectively inhibit G proteins that are sensitive to Pertussis toxin (**C**), ATPA-induced inhibition of primary sensory afferents was markedly reduced, consistent with a model in which GluK1 KAR-mediated modulation of glutamate release requires the activation of a G protein. The data shown in B-C are taken from Rozas et al. (2003) ([80]). For additional details, please refer to that publication.

Kainate receptor activation suppresses N-type voltage-gated Ca^2^⁺ currents through a reversible, G protein–dependent, metabotropic mechanism that is independent of their ion channel activity (Fig. 3C). In DRG neurons lacking ionotropic responses to kainate, the inhibitory effect on VGCC currents still persisted, indicating that the permeation through the KAR ion channel is not required for non-canonical signaling. Indeed, kainate is able to inhibit N-type VGCCs even in sodium-free extracellular conditions, where ion channel gating is severely compromised [14]. Moreover, the magnitude of kainate-induced inhibition of K⁺-evoked calcium responses does not correlate with the extent of kainate-induced Ca^2^⁺ elevation, demonstrating the independence of these two signaling modes [80]. Ionotropic and metabotropic KARs are spatially segregated across dissociated DRG neurons, often localizing to distinct subcellular regions. Confocal calcium imaging revealed that the sites where KARs inhibit VGCC activity (Fig. 3D) do not always coincide with those where kainate evokes a detectable calcium influx (Fig. 3E). Certain regions showed strong inhibition of K⁺-evoked calcium signals without a corresponding kainate-induced calcium increase, while other areas exhibited robust ionotropic responses but minimal inhibition (Fig. 3F). This spatial segregation illustrates the notion that metabotropic and ionotropic signaling by KARs occur in different cellular compartments, reinforcing their functional independence. Indeed, KARs suppress calcium influx through VGCCs in a concentration-dependent manner, even at concentrations that are incapable of producing any depolarization. ATPA, a selective agonist for GluK1-containing receptors, similarly depresses K⁺-evoked calcium responses. This effect is abolished in GluK1-deficient mice but remains in GluK2 knockouts, indicating a critical and non-redundant role for GluK1 in metabotropic signaling in DRG neurons. Nonetheless, the potential involvement of auxiliary subunits such as GluK4 or GluK5 remains unresolved, as these require co-expression with low-affinity subunits for surface expression (see [80]). At the synaptic level, GluK1-containing KARs modulate glutamate release from primary afferents in the spinal cord [80]. ATPA application reduces excitatory postsynaptic potentials in spinal cord slices via a G-protein–dependent mechanism, as shown by its sensitivity to N-ethylmaleimide (NEM), a G-protein inhibitor (Fig. 4). This presynaptic inhibition mirrors observations in the hippocampus, where kainate reduces presynaptic Ca^2^⁺ influx and suppresses excitatory transmission in CA1 [88] and CA3 [89].

Whether ionotropic and metabotropic functions coexist within the same receptor complex or represent separate receptor populations remains unresolved. Some GluK1 splice variants lack functional channel activity when expressed alone but may gain function through heteromeric assembly, suggesting a dynamic and context-dependent signaling repertoire. It is also possible that KARs acquire metabotropic functionality through interactions with adaptor proteins or G protein–coupled receptor-like components, similar to hybrid complexes observed between ionotropic and G-protein–coupled receptors described for dopamine and NMDA/GABAA receptors [90, 91]. Proteomic data from Rutkowska-Wlodarczyk *et al.* (2015) [92] have provided new insights into this signaling paradigm by identifying GluK1 as a central player in noncanonical KAR signaling. Their analysis revealed a broad GluK1 interactome, including proteins involved in vesicle trafficking, cytoskeletal organization, and signal transduction—such as 14-3-3, CaMKII, Rab3a, synapsin II, and Basp1. A key finding of this study is the direct interaction between GluK1 and the α subunit of Go protein. This coupling was validated through bioluminescence resonance energy transfer (BRET) assays, and the activation of Go signaling was shown to be specific to GluK1, absent in GluK1-deficient mice [92]. These results provide a mechanistic basis for previously reported metabotropic effects of KARs, especially in DRG neurons. Importantly, the GluK1b isoform, which possesses a longer C-terminal domain, was identified as both necessary and sufficient for Go activation, establishing GluK1 as the subunit primarily responsible for linking glutamate binding to downstream G-protein-mediated signaling [92]. Remarkably, KARs also modulate neuronal development in DRG neurons in a bidirectional manner that is mediated by the activation of two distinct signaling pathways. Weak activation of KARs delays maturation and promotes neurite outgrowth, whereas moderate to strong KAR activation promotes the maturation of neurons while limiting neuritic growth [93].

In sum, GluK1-containing KARs play a dual role in DRG neurons: they function as classical ion channels and as metabotropic modulators of calcium dynamics and neurotransmitter release. This multifaceted signaling capacity positions KARs as pivotal regulators of sensory input and highlights their potential as therapeutic targets for modulating nociceptive processing.

## Functional Characteristics of DRG Neuronal Populations Expressing KARs

The populations of DRG that most abundantly express *Grik1* are Th, Mrgprd, Mrgpra3+Mrgprb4, and Mrgpra3+TrpV1. Recent studies in mice have described distinct functional properties of Th neurons (identified as C-LTMRs by [20]), Mrgprd+ and Mrgprb4+ neurons (corresponding to the Mrgprd and CGRP-θ2 clusters, respectively, in [20]), as well as Mrgpra3+ neurons (identified as the CGRP-θ1 cluster). These populations of neurons have been functionally profiled regarding their mechanical and thermal sensitivity characteristics, providing new clues on the roles that KARs may play in sensory physiology.

Th neurons (expressing tyrosine hydroxylase) are a major source of *Grik1* expression (96% of Th neurons express *Grik1*) and are characterized by exquisitely low mechanical thresholds (<10 mN), with most neurons responding to light mechanical forces around 15 mN. Their response profile demonstrates graded activation across low-force ranges and plateaus at moderate forces (20–40 mN), clearly defining them as classical low-threshold mechanoreceptors. Th neurons display robust responses to both air puff and cotton swab stroke, consistent with the idea that LTMRs saturate in the innocuous range. Functionally, Th neurons display an intermediate adaptation rate: they show robust onset and offset responses with a slowly decaying calcium signal during sustained mechanical stimuli, consistent with their role in encoding gentle, dynamic touch. These neurons are also selectively responsive to innocuous cooling, exclusively during temperature decreases, but show limited or no activation by noxious heat or high-intensity mechanical stimuli [20]. Consistent with these findings, Li *et al.* (2016) [23] also identified a DRG neuronal population marked by strong Th expression (named the C3 cluster) and functionally characterized it using *in vivo* whole-cell patch-clamp recordings of single neurons in the L5 DRG of mice. Although a subset of these neurons responded to high-intensity mechanical stimuli such as pressure or pinch, the majority were selectively activated by innocuous tactile stimuli (e.g., brushing) and never responded to heat. This physiological profile further supports the designation of Th neurons as dedicated LTMR, specialized for the detection of non-painful touch, often referred to as affective or emotional touch, and innocuous cooling. These neurons may function as pain modulators rather than primary transmitters of nociceptive pain. In contrast, Mrgprd+ (98.2% expressing *Grik1*) and Mrgprb4+ (90% expressing *Grik1*) neurons exhibit broad dynamic ranges in mechanical sensitivity, with thresholds typically between 5–20 mN but extending into higher force domains. This mechanosensitivity places them within the C-HTMR or nociceptor category. In addition to a gentle stroke, both subtypes showed slowly adapting responses to sustained mechanical indentation and are robustly activated by intense mechanical stimuli such as pinch. Notably, these neurons are polymodal since Mrgprd and Mrgprb4 neurons also respond to noxious heat (>45 °C) and are involved in encoding thermal pain. The expression of TRPM3, TRPV2, and TRPA1 channels in these populations may contribute to their thermal sensitivity [20].

Mrgpra3+ (76.5% expressing *Grik1*) neurons share overlapping functional characteristics with Mrgprd and Mrgprb4 populations. They exhibit higher mechanical thresholds (20–40 mN), sustained calcium activity under prolonged stimulation, and pronounced responses to warmth (~40 °C), falling into the HTMR or the nociceptor category as well. Importantly, this subtype also expresses TRPV1 and a suite of itch-related receptors, supporting its dual role in thermosensation and pruriception. Subsets of both Mrgprb4 and Mrgpra3 neurons were also activated transiently by relative increases in temperature, particularly from cold to neutral ranges, suggesting sensitivity to dynamic thermal changes [20]. Li *et al.* (2016) [23] further characterized these populations transcriptomically and functionally. Using single-cell RNA sequencing, they identified Mrgprd+ neurons as part of their C5 cluster, and Mrgpra3+ neurons within their C4 cluster, which includes a subcluster (C4-2) distinguished by co-expression of *Mrgprb4*. Through *in vivo* whole-cell patch-clamp recordings in L5 DRG neurons of mice, the authors demonstrated that these neuronal groups are generally responsive to noxious mechanical stimuli and heat, classifying them functionally as mechanoheat nociceptors (MHNs). Importantly, they also observed moderate sensitivity to innocuous mechanical stimulation, such as pressure. While most Mrgpra3+ and Mrgprb4+ neurons responded robustly to pinch, Mrgprb4+ cells are also sensitive to gentle touch, such as affective or pleasant touch. These neurons are additionally itch-sensitive neurons, which led the authors to propose that these neurons are polymodal MHNs, capable of integrating pruritic, thermal, and mechanical inputs with distinct stimulus preferences.

Collectively, these findings indicate that *Grik1* is expressed in two functionally distinct DRG neuronal populations: one associated with non-noxious sensory modalities, such as gentle touch (Th neurons and part of Mrgprb4+ cells), and another closely linked to the detection of noxious mechanical, thermal, and pruritic stimuli, including the Mrgprd, Mrgpra3+Mrgprb4, and Mrgpra3+Trpv1 subtypes. This distribution positions GluK1 as a potential key modulator involved in mechanisms of both nociception and normal somatosensation, underscoring its relevance as a therapeutic target for the regulation of nociceptive signaling.

Although also expressed by a fraction of Mrgprd neurons (56%), *Grik2* is mainly expressed by two different neuronal populations (Fig. 2). On one side, Pvalb neurons are classically associated with proprioception. On the other hand, Trpm8+ neurons are characterized by small-diameter, non-peptidergic neurons with minimal overlap with IB4 and CGRP markers [20]. Functionally, Trpm8 neurons are mechanosensory-inactive, exhibiting no calcium responses to a wide range of mechanical stimuli, including step indentations, pinch, or Von Frey filaments. In contrast, these neurons display pronounced thermal sensitivity, with high sensitivity to cool, even moderately cool temperatures. They are robustly activated by mild decreases in temperature and remain responsive throughout sustained cooling epochs. A notable fraction (~40%) exhibits reduced activity upon warming, suggesting tonic activation at physiological skin temperatures and a role in encoding relative thermal changes. Unlike nociceptive subtypes that respond to noxious cold, Trpm8+ neurons contribute to innocuous cool detection, and their function is tightly associated with the expression of the Trpm8 ion channel, confirming their specialized role in cold thermosensation rather than nociception.

These recent data call for a reassessment of the potential contributions of KARs to sensory physiology. Along this line, functional studies in mice have established a specific role for the KAR subunit GluK2 (encoded by *Grik2*) in the detection of cold, but not cool, temperatures. Behavioral assays show that GluK2 KO mice retain normal responses to innocuous cool stimuli (~18 °C), while selective loss of GluK2 in DRG neurons impairs behavioral responses to cold (<15 °C) and cold-induced pain (0 °C) [94]. Notably, the combined deletion of *Grik2* and *Trpm8* results in profound deficits in cold avoidance and nociception, revealing non-redundant roles for these receptors. Calcium imaging and electrophysiological recordings further support the involvement of GluK2 in cold-specific DRG neurons, which are distinct from Trpm8+ cool-sensing populations [94]. This is supported by the fact that *Grik2* is expressed in approximately 68% of the Trpm8 neuron population, but only 15% of the Grik2-expressing cells express *Trpm8*, as defined by Bhuiyan *et al.* (2024) [25] in DRG neurons, indicating that *Grik2* could serve roles in cold sensation beyond Trpm8. This divergent expression also highlights an important distinction: while *Grik2* is transcriptionally present in most cool-sensing neurons, functional studies demonstrate that GluK2 is not required for innocuous cool perception. In contrast, GluK2 is essential for cold-specific and cold-pain responses, which are mediated by a distinct population of cold-sensitive, Trpm8-independent neurons. These findings underscore the importance of integrating transcriptomic and physiological data when interpreting the role of ionotropic receptor subunits in somatosensory function. Moreover, the expression of *Grik2* in other neuronal subtypes, such as Pvalb*,* Mrgprd, and Sst neurons, raises the possibility that GluK2 may exert its role in cold nociception through alternative neuronal sensors.

Although the harmonized atlas developed by Bhuiyan *et al.* (2024) [25] successfully integrates DRG neuronal subtypes across species and datasets, it is important to note that a few rare subtypes, such as Mrgpra3+Mrgprb4, Mrgpra3+Trpv1, and Calca+Oprk1, were not uniformly detected across all studies. Notably, these populations, which are clearly present in mouse DRG, are absent from current human snRNA-seq datasets. However, evidence from in situ hybridization studies and human whole-soma RNA-seq data suggests that at least Mrgpra3+Mrgprb4, Mrgpra3+Trpv1 likely exist in human DRG neurons [36, 95, 96]. The lack of detection of these groups in some data sets may be due to the technical limitations of snRNA-seq, which generally captures fewer transcripts per cell compared to scRNA-seq. Given that key defining transcripts for rare neuronal identities may be underrepresented in nuclear RNA, incorporating higher-resolution scRNA-seq data, particularly from human and other large-mammal ganglia, will be critical to further refine the classification of rare subtypes and enhance the translational utility of future reference atlases [25].

**Fig. 5:**
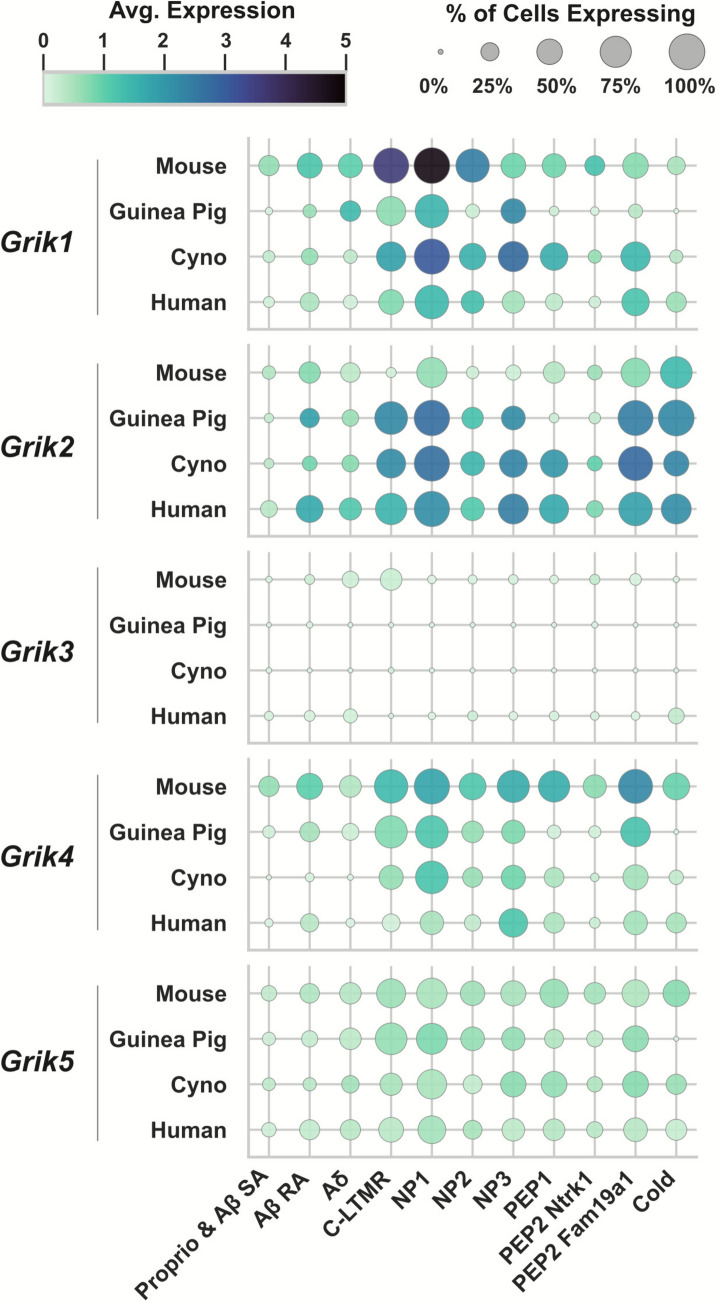
Dot plot showing the average expression (color intensity) and proportion of expressing cells (dot size) for individual KAR subunits (rows) across clusters defined in mouse, guinea pig, cynomolgus monkey, and human DRG neuronal subpopulations (columns), based on single-cell RNA sequencing data from Jung *et al*. (2023) ([27]). *Grik1* and *Grik2* are abundantly expressed in sensory neuron subtypes associated with nociception and mechanosensation. *Grik1* is prominently expressed in non-peptidergic (NP) nociceptors and thermosensitive neurons, consistent with previous reports of its functional role in peripheral pain transmission. In contrast, *Glik4* and *Grik5* show more restricted expression patterns. PEP and NP denote peptidergic and non-peptidergic neurons, respectively. These data indicate a possible evolutionary divergence in KAR engagement across species, with *Grik2* assuming a more dominant role in primates. Data taken from the SpeciesDRGAtlas website (http://research-pub.gene.com/XSpecies DRGAtlas/). See [27].

Comparative cross-species transcriptomic analyses of DRG neurons from mouse, guinea pig, cynomolgus monkey, and human, as developed by Jung *et al.* (2023) [27], provide a resource for exploring evolutionary aspects of sensory neuron diversity. Their openly accessible dataset enables direct examination of KAR subunit (*Grik1–Grik5*) expression across homologous DRG neuronal populations, within a unified classification framework. Unlike the harmonized atlas, which integrates data across species and studies into a single composite reference, this species-resolved approach preserves interspecies variation, although with a lower power. Yet it allows the identification of shifts in KAR subunit expression patterns between species that are not readily detectable in the harmonized atlas, where integration may obscure these biologically relevant differences. Thus, this analysis revealed distinct and partially conserved expression patterns of KAR genes (*Grik1–Grik5*) across species and neuron subtypes. The dot plot in Figure 5 illustrates the average expression (color intensity) and proportion of expressing cells (dot size) of each KAR subunit across subtype clusters defined in mouse, guinea pig, cynomolgus monkey, and human DRGs. Specifically, *Grik1* and *Grik2* emerged as the most abundantly expressed genes in sensory neuron subtypes associated with nociception and mechanosensation. *Grik1* shows robust expression in mice across NP1 and NP2 groups, which Bhuiyan *et al.* (2024) [25] classify as part of Mrgprd, Mrgpra3+Trpv1, and Mrgpra3+Mrgprb4 populations, and Th C-LTMRs. In the cynomolgus monkey, the overall pattern of *Grik1* expression appears broadly conserved relative to mouse, although expression levels slightly decrease in the NP1 population and increase in the Sst NP3 group. In humans, *Grik1* expression declines further across all subtypes; however, NP1 neurons continue to show the highest relative expression, indicating partial conservation of subtype specificity despite the overall reduction in expression. In contrast, *Grik2* shows robust expression in primate DRG neurons, including human, particularly in NP1, Sst, NP3, Cold Trpm8, and Fam19a1 PEP2 neurons. According to Bhuiyan *et al.* (2024) [25] nomenclature, this latter group is formed by Calca+Bmpr1b and Calca+Smr2. This shift suggests a possible evolutionary divergence in KAR engagement across species, with *Grik2* assuming a more dominant role in primates. These differences highlight the importance of considering species-specific expression when evaluating the functional or therapeutic relevance of KARs.

Notably, fast-conducting proprioceptive and mechanoreceptive neurons display minimal KAR expression, indicating that these receptors are unlikely to participate in rapid sensory transmission. Instead, their expression is preferentially enriched in polymodal sensory neurons, which respond to mechanical, thermal, and chemical stimuli. This subtype-specific distribution suggests that KARs may play a more prominent role in slower or modulatory aspects of sensory processing -such as synaptic integration or excitability thresholds, particularly in relation to pain and thermal sensation.

In summary, the expression of KAR subunits in DRG neurons is subtype-specific, functionally compartmentalized, and evolutionarily dynamic. The enrichment of *Grik1* and *Grik2* in nociceptors and thermoreceptors suggests that KAR signaling might contribute to excitability thresholds or synaptic integration in pain pathways, highlighting them as potential pharmacological targets. Moreover, based on the expression patterns described above, KAR subunits appear to be intimately involved in pain processing. Specifically, *Grik1* is predominantly associated with neuronal subtypes responsive to mechanical and heat noxious stimuli, whereas *Grik2* shows preferential enrichment in populations involved in noxious cold sensing. This distribution suggests a differential but complementary role of these subunits in shaping the modality-specific encoding of painful stimuli. Altogether, these expression patterns enrich our understanding of glutamatergic signaling diversity in the somatosensory system and provide a molecular framework for guiding translational studies in pain and sensory modulation. However, interspecies differences in subunit expression and receptor composition must be carefully considered before delineating translational applications.

## Functional Kainate Receptors in Pain-Associated Pathways

### Kainate Receptors in Primary Sensory Neurons

**Fig. 6:**
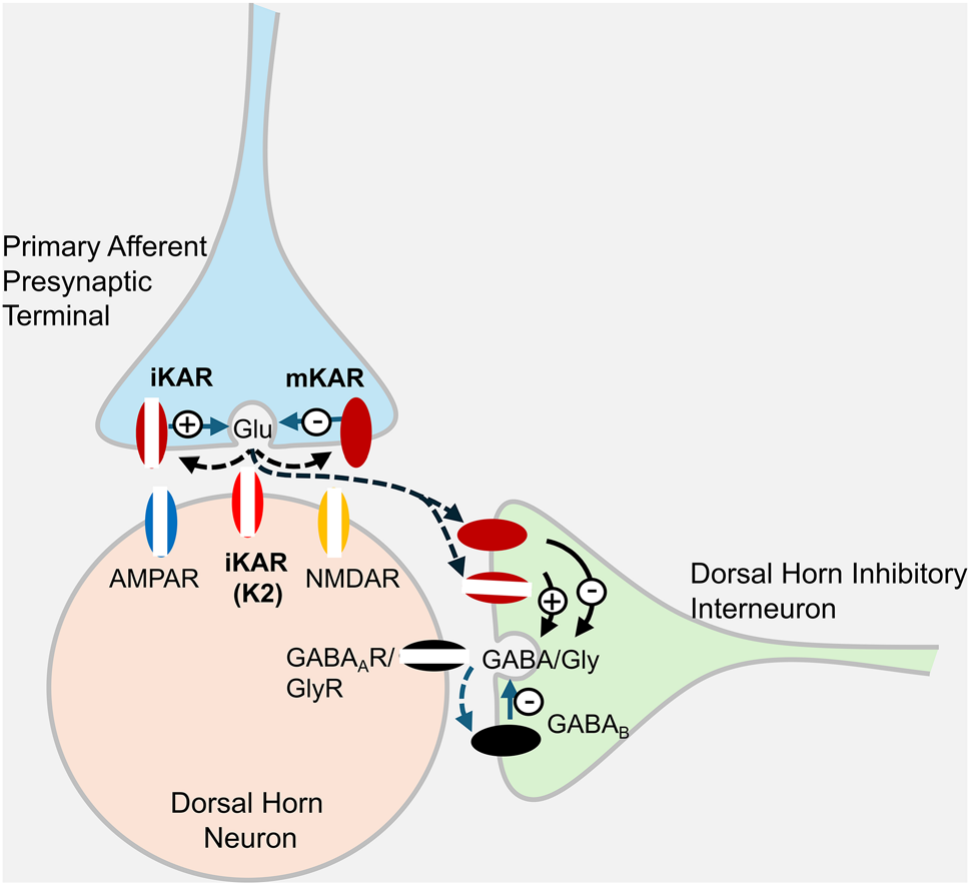
KARs modulate both excitatory and inhibitory transmission in the sensory to dorsal horn neurons. Schematic representation of the proposed distribution of KAR subunits at primary afferent synapses in the spinal cord. Presynaptic ATPA-sensitive KARs on primary afferents bidirectionally regulate glutamate release [85], whereas ATPA-insensitive postsynaptic KARs (likely GluK2) in dorsal horn neurons facilitate nociceptive transmission [105]. Glutamate spillover can also recruit presynaptic KARs on inhibitory terminals, modulating glycine and/or GABA release and indirectly reducing inhibition through GABAB autoreceptors. Both ionotropic (iKARs) and metabotropic (mKARs) KARs are thought to involve ATPA-sensitive GluK1 subunits [80].

Although antibodies recognizing different KAR subunits are missing, presumptive KARs have been localized in peripheral nerve terminals within the skin of rats, where they are proposed to serve as detectors of glutamate released in response to tissue injury [97, 98]. Additionally, these receptors are also located at presynaptic terminals of primary afferent neurons in the dorsal horn of the spinal cord, where they are thought to act as autoreceptors modulating neurotransmitter release (Fig. 4 and 6) [85, 99–101]. The physiological and pharmacological properties of KAR-mediated currents in isolated small-diameter DRG neurons closely resemble those of recombinant homomeric GluK1 receptors expressed in heterologous systems [18, 52]. Complementary studies in HEK cells suggest that GluK1/GluK5 complexes mimic native desensitizing DRG responses more accurately, as they lack the rapid desensitization typically observed in GluK2/GluK4 or GluK2/GluK5 combinations [52, 61, 102]. High-resolution single-cell transcriptomic profiling illustrates a strong convergence between electrophysiological, histological, and transcriptomic approaches, supporting the view that KARs in DRG neurons are strategically positioned to influence nociceptive and thermoceptive signaling.

Other KAR subunits, such as GluK2 and GluK3, are also detected in mice DRG neurons, albeit at relatively lower levels. It is unlikely that these latter subunits alone contribute to the formation of functional KARs, as studies on dissociated DRG neurons from GluK1 knockout mice have shown a complete absence of currents in response to kainate [80, 99]. These findings indicate that GluK1 is essential for the formation of functional receptors in DRG cells. While the deletion of GluK2 does not affect the amplitude of kainate-evoked current, differences in desensitization kinetics between wild-type and GluK2^-/-^ cells suggest that GluK2 might contribute to KARs in DRG cells [99], although not playing a fundamental role in these types of neurons. These data also argue against a significant contribution of AMPA receptors, which are also sensitive to kainate, although their presence in DRG neurons has been confirmed by recent transcriptomic data. Further evidence for the pivotal role of GluK1 comes from studies utilizing GluK1-selective agonists and antagonists. The specific agonist for GluK1-containing KARs, ATPA, has been shown to reduce DRG–dorsal horn synaptic transmission to a similar extent as kainate [80, 99]. However, ATPA does not affect synaptic transmission between cultured dorsal horn neurons [85], corroborating, once again, that the GluK1 subunit is a fundamental and exclusive part of KARs in DRG neurons. The study using specific antagonists for the GluK1 subunit, such as LY382884, also supports that this subunit is the main one in DRG neurons. Therefore, it seems well-established that GluK1 is an essential subunit for assembling functional KARs in DRG neurons. It is worth noting that the efficacy of these agonists is higher for native receptors from DRG neurons than for recombinant homomeric GluK1 receptors, suggesting the involvement of other subunits or interacting proteins in the receptor functionality.

### Kainate Receptors Underlying DRG to Spinal Cord Neuron Transmission

Synaptic communication between DRG neurons and those in the dorsal horn of the spinal cord involves all three major ionotropic glutamate receptor types: NMDA, AMPA, and KARs. In the spinal cord, KARs fulfill a dual function in sensory transmission. In dorsal horn neurons, they mediate a part of synaptic transmission and regulate postsynaptic membrane excitability; at presynaptic terminals of DRG fibers, they play a critical role in regulating sensory transmission by modulating glutamate release [80, 85, 101]. Indeed, activation of these receptors with kainic acid or the GluK1-selective agonist ATPA significantly reduces both NMDA-mediated evoked excitatory postsynaptic currents (EPSCs) and miniature EPSC frequency in dorsal horn neurons, as observed by DRG stimulation in both cocultures and spinal cord slice preparations from rats or mice [80, 85, 99, 101]. Kainate application normally suppresses both DRG-to-spinal and spinal-to-spinal excitatory transmission [80, 85], an effect mediated by presynaptic KARs located in DRG neurons and dorsal horn neurons, respectively. However, when either GluK1 or GluK2 subunits were deleted, the suppressive effect of kainate was unexpectedly reduced. To explain this, Kerchner and colleagues (2002) [99] suggested that electrical stimulation in their coculture preparations may have activated not only DRG terminals but also adjacent dorsal horn excitatory neurons or axons. Therefore, in GluK1- or GluK2-deficient cocultures, kainate could only act on one component of the circuit, either the presynaptic DRG (*via* GluK1) or the postsynaptic dorsal horn (*via* GluK2), leading to a partial reduction in EPSCs (Fig. 6). This interpretation is supported by the observation that kainate failed to affect EPSCs in mixed cocultures of GluK1^-/-^ DRGs cells and GluK2^-/-^ dorsal horn neurons [99]. Furthermore, ATPA selectively activates KARs in cultured DRG neurons but fails to activate those in cultured dorsal horn neurons [85], suggesting that ATPA-mediated inhibition of synaptic transmission is unlikely to involve direct postsynaptic mechanisms. This modulatory effect of kainate and ATPA is abolished in animals deficient of GluK1 subunits, even when cocultured with wild-type or GluK2-deficient dorsal horn neurons [99, 103]. Additionally, the selective GluK1 antagonist LY382884 blocked the effect of kainate in cocultures derived from GluK2-deficient mice [99]. Such a presynaptic modulation of synaptic transmission may be mediated by either primary afferent depolarization [104] or, alternatively, through a metabotropic signaling cascade involving G proteins, which leads to a reduction in calcium channel activity [80]. Interestingly, since he presynaptic modulation of glutamate release by KARs exhibits a bidirectional pattern: low concentrations of kainate facilitate glutamate release, while higher concentrations exert an inhibitory effect [101]. It is possible that ionotropic and metabotropic KARs have different ligand affinities. Therefore, KARs may facilitate glutamate release when activated by a relatively low dose of their agonists and inhibit it when activated by higher concentrations. This dual modulation suggests that KARs may act as fine-tuning regulators of synaptic strength in a dose-dependent manner.

### Kainate Receptors in Dorsal Horn Neurons

*In situ* hybridization and transcriptomic datasets have shown that transcripts encoding KAR subunits are distributed across multiple laminae of the spinal dorsal horn, with *Grik1* being the most abundantly expressed, whereas *Grik4* is found at much lower levels. Functional evidence from electrophysiological recordings demonstrates that pharmacologically isolated KAR-mediated excitatory postsynaptic currents (EPSC_KA_) can be evoked in lamina II neurons upon stimulation of primary sensory afferents [105]. These currents exhibit distinct biophysical properties: they show slower gating kinetics, reduced peak amplitudes, and prolonged decay phases compared to AMPA receptor-mediated EPSCs recorded at the same synapses. Notably, EPSC_KA_ are preferentially recruited by high-intensity stimulation, indicating their selective activation by nociceptive Aδ and C fibers. In contrast, low-intensity stimulation fails to evoke EPSC_KA_ but reliably triggers EPSC_AMPA_, pointing to a functional segregation of ionotropic glutamate receptor types across afferent pathways. This distribution suggests that postsynaptic KARs are predominantly localized at synapses receiving input from high-threshold nociceptive fibers, while AMPARs are enriched at synapses driven by low-threshold afferents [105]. Furthermore, the apparent absence of KAR-mediated responses at postsynaptic sites in lamina I, typically targeted by C-fiber terminals [106], appears contradictory. However, neurons expressing Mrgprd, which show the highest proportion of *Grik1* expression, project predominantly to lamina IIi of the dorsal horn, overlapping with the IB4-binding. Similarly, other *Grik1*-enriched populations, including Mrgpra3+ and Mrgprb4+ subgroups, also terminate largely in lamina IIi, reinforcing the idea that KARs are strategically positioned within nociceptive circuits. In contrast, C-LTMRs (Th subtype), where *Grik1* expression is also robust, extend their terminals slightly deeper into lamina IIiv.

The distinct temporal dynamics of EPSC_KA_, particularly their prolonged decay, may support extended synaptic integration during high-frequency stimulation, potentially contributing to amplification of nociceptive signaling under persistent or pathological pain conditions. Supporting this, retrograde labeling from thalamic relay neurons has confirmed the presence of functional KARs in projection neurons located in lamina II, suggesting a role for these receptors in ascending nociceptive transmission [105]. Altogether, these findings underscore the strategic placement of spinal KARs within dorsal horn circuits and their potential involvement in modulating excitatory drive and central sensitization in chronic pain states.

Studies in cultured murine dorsal horn neurons from KO animals [99, 101] indicated that KARs comprise both GluK1 and GluK2 subunits. While the GluK1 deletion had minimal impact on kainate-evoked current densities compared to wild-type, the absence of GluK2 significantly reduced KAR-mediated currents in a subset of dorsal horn neurons, while having no effect on others. These findings indicate that GluK2 plays a more important role than GluK1 in the formation of functional KARs in dorsal horn neurons [99]. Despite this, it remains unclear whether these subunits predominantly form heteromeric receptors or whether distinct populations of homomeric receptors also exist. Evidence supporting the involvement of GluK1 comes from the observation that GluK1 antagonists effectively block the initial current response upon agonist application in wild-type neurons [99]. The lack of response to ATPA in dorsal horn neurons indicates that these neurons express KARs with a low proportion of GluK1 subunits, if any. This could explain why eliminating GluK1 had little effect on the overall KAR-mediated current density. Collectively, although the contribution of other subunits cannot be ruled out, these findings suggest that while some dorsal horn neurons express heteromeric receptors composed of both GluK1 and GluK2, others may rely exclusively on GluK2-containing KARs [99, 101].

KARs are not limited to excitatory synapses; they have also been identified at inhibitory synapses. Kerchner *et al* (2001) [107] demonstrated that inhibitory neurons in the dorsal horn express presynaptic KARs, that modulate the release of GABA and glycine, and this modulation is in intimate relationship with glutamate release (Fig. 6). Therefore, KARs appear to play a unique role among ionotropic glutamate receptors in regulating the release of inhibitory transmitters in the dorsal horn. Evidence indicates that KARs are located at inhibitory presynaptic terminals, where their activation directly promotes vesicular release of GABA and glycine independently of somatic depolarization [99, 107]. This function of presynaptic KARs in inhibitory neurons follows a bidirectional mode of action, facilitating evoked inhibitory postsynaptic currents at low agonist concentrations but suppressing them at higher concentrations through a mechanism likely dependent on presynaptic GABA_B_ autoreceptors. Moreover, selective GluK1 antagonists such as LY382884 and LY293558 significantly blocked kainate-evoked currents in dorsal horn neurons, and GluK1 deletion alone partially reduced kainate current density and did not abolish presynaptic modulation of inhibition, indicating that this subunit is partially responsible for the maintenance of inhibitory control in the dorsal horn [99]. By comparison, GluK2 deletion produced a marked reduction in kainate current density, with approximately half of the neurons rendered unresponsive and the remaining cells showing smaller currents relative to wild-type or GluK1-deficient neurons. Nevertheless, kainate-mediated suppression of evoked inhibitory transmission persisted in GluK2 knockout.

Taken together, these studies suggest that, similarly to hippocampal interneurons [108], both GluK1 and GluK2 contribute to functional KARs in dorsal horn inhibitory interneurons, most likely in the form of heteromeric assemblies. Neither subunit alone is indispensable for presynaptic modulation, in contrast to dorsal root ganglion neurons, where GluK1 is critical.

### Kainate Receptors in Thalamocortical and Cortical Circuits

Ascending nociceptive signals from the dorsal horn are transmitted to the ventral posterolateral nucleus of the thalamus via the spinothalamic tract, a major pathway in pain perception. In situ hybridization and immunostaining studies have demonstrated high expression of GluK1, GluK2, GluK3, and GluK5 mRNAs in the cortex, while GluK4 expression remains low during postnatal days [109, 110]. Within the thalamus, KAR subunit expression displays region-specific patterns, with subtypes such as GluK1 exhibiting prominent localization in relay-related nuclei [109, 111, 112]. Functionally, GABAergic reticular neurons exert inhibitory control over thalamic relay neurons, and this inhibition is regulated by presynaptic GluK1-containing KARs, which modulate GABA release. Evidence from rat ventrobasal thalamus recordings indicates that activation of these receptors suppresses GABAergic transmission, potentially facilitating thalamocortical relay activity through disinhibition [113, 114]. In support of this view, GluK1-selective antagonists have been shown to attenuate sensory-evoked responses—such as those elicited by whisker stimulation—suggesting that KARs contribute to thalamic excitability and could serve as therapeutic targets in conditions involving central hyperexcitability, including chronic pain. Additional complexity arises from corticothalamic circuits, where KARs at presynaptic terminals exhibit divergent effects: GluK1-containing receptors suppress glutamate release at relay neuron synapses, while KARs lacking GluK1 enhance glutamate release at reticular neuron terminals [115]. This duality implies a layered regulatory mechanism over thalamic excitability, combining direct and indirect modulation of relay neuron output.

KARs also play diverse roles in pain-relevant cortical regions. In the anterior cingulate cortex (ACC), a key structure in affective and cognitive aspects of pain, both pre- and postsynaptic KARs are present [4, 116–119]. Experiments employing mice lacking GluK1 or treated with GluK1-selective antagonists have demonstrated that presynaptic GluK1-containing KARs contribute to synaptic transmission and plasticity in the adult ACC and insular cortex [120, 121]. Notably, presynaptic GluK1-containing KARs enhance GABA release from local interneurons targeting pyramidal cells in layers II/III, contributing to the regulation of tonic inhibition and network excitability [100]. Postsynaptically, EPSCs mediated by KARs in the ACC require both GluK1 and GluK2, as demonstrated by using subunit-specific knockout models [122]. Glutamatergic synaptic transmission from the insular cortex projecting to the basolateral amygdala has been found to be involved in the process of pain empathy [123, 124]. Finally, although less studied, the periaqueductal gray —a midbrain region integral to descending pain control— also expresses KARs. *In vitro* studies in dissociated periaqueductal gray neurons have shown that presynaptic GluK1-containing KARs regulate GABA release, potentially modulating the output of this critical hub for endogenous analgesia [125].

Altogether, these findings emphasize that KARs are expressed and functionally active across multiple levels of the pain neuraxis, from the spinal cord to thalamic and cortical circuits.

## Kainate Receptors as Possible Therapeutic Targets

Despite the poorly selective pharmacology developed for KARs, accumulating evidence from preclinical and clinical studies highlights a pivotal role of KARs in modulating nociceptive transmission. Multiple studies have demonstrated that pharmacological blockade or desensitization of KARs produces robust antinociceptive effects across various experimental pain models. Intrathecal administration of the nonselective AMPA/KAR antagonist CNQX produced dose-dependent antinociception in both the tail-flick and hot-plate tests. In contrast, the AMPA-selective antagonist SYM2206 affected only the tail-flick reflex at high doses. The high-affinity desensitizing agonist (2S,4R)-4-methylglutamate (SYM2081) potently activates and desensitizes GluK1- and GluK2-containing KARs, rendering them functionally silent. SYM2081 produced significant antinociceptive effects in both assays, supporting a functional role of KARs in spinal pain signaling [105]. In neuropathic pain models, SYM2081 significantly attenuated the enhanced nociceptive responsiveness to both thermal and mechanical stimuli, indicating that sustained KAR desensitization may modulate both acute and neuropathic pain [126]. Subsequent work confirmed that systemic or intrathecal administration of SYM2081 prevents and reverses capsaicin- and carrageenan-induced mechanical and thermal hyperalgesia, with effects localized to the central nervous system rather than peripheral sites [127].

Further pharmacological evidence for KAR involvement in nociceptive processing came from studies using formalin-induced pain models in rats. In this model, the mixed AMPA/KAR antagonist NBQX produced antinociception but caused ataxia, while the AMPA-selective antagonist LY300164 was ineffective at non-ataxic doses. The GluK1-selective antagonist LY382884, however, produced robust analgesia without motor impairment, confirming a specific role of GluK1-containing KARs in persistent inflammatory pain [72]. Similarly, inflammation-induced hyperalgesia was attenuated by NBQX, NS-102, and LY382884, accompanied by selective upregulation of GluK1 and GluK2 (predominantly the unedited GluK2(Q) isoform), suggesting transcriptional sensitization of KAR signaling during inflammation [128].

Consistent with these findings, additional evidence supporting the analgesic potential of kainate receptor modulation comes from studies with NS1209, an AMPA/GluK1-selective antagonist (but see [129]). NS1209 produced strong analgesic effects in both acute (hot-plate) and persistent (formalin) pain models, outperforming NBQX, which was ineffective in these assays. In neuropathic pain models, NS1209 reduced mechanical and cold hypersensitivity without impairing motor function, supporting analgesic efficacy at non-ataxic doses [130]. Similarly, oral administration of GluK1-selective antagonists LY467711 and LY525327 reduced late-phase formalin responses, carrageenan-induced thermal hyperalgesia, and capsaicin-induced mechanical hyperalgesia [131, 132]. The selective GluK1 functional antagonist MSVIII-19 also showed dose-dependent antinociception after intrathecal delivery, attenuating formalin-, inflammation-, and nerve injury–induced hypersensitivity without affecting motor performance or visceral pain, again highlighting the therapeutic potential of spinal GluK1 receptor blockade in chronic pain [133].

In contrast to the analgesic effects produced by KAR antagonists, peripheral activation of AMPA/KARs has been associated with the induction of hyperalgesia. Systemic (intraperitoneal or subcutaneous) injection of kainic acid produced persistent thermal and mechanical hyperalgesia in rodents, whereas the AMPA/KAR antagonist CNQX abolished these effects. In contrast, intrathecal kainate administration failed to induce hyperalgesia, suggesting that peripheral rather than spinal KARs—likely located on nociceptive C-fiber terminals—mediate these responses [134].

However, studies using selective GluK1 ligands show that ATPA reduces nociceptive reflexes in hemisected spinal cord preparations from neonatal rats in vitro but is ineffective in in vivo models. In contrast, the GluK1-selective antagonists LY294486 and LY382884 attenuated nociceptive responses in all tests, though only at doses that also influenced AMPA receptor–mediated activity. These results suggest that while GluK1 receptors contribute to spinal nociceptive transmission, their selective blockade provides limited analgesic efficacy in acute pain models [135]. Similarly, intrathecal ATPA administration produced dose-dependent increases in hindpaw withdrawal latencies to both thermal and mechanical stimulation, confirming a spinal site of action [136]. Genetic evidence reinforces the pharmacological data, showing that GluK1 and GluK2 subunits contribute to distinct behavioral domains. Mice lacking GluK1 exhibited significant reductions in capsaicin-induced and inflammatory pain, whereas GluK2 knockout mice showed normal nociception. These findings indicate that GluK1-containing receptors modulate nociceptive transmission more effectively than GluK2 receptors, underscoring subtype-specific functional roles [137].

Clinical attempts to translate these findings have shown moderate success. The AMPA/kainate receptor antagonist LY293558 (tezampanel) reduced capsaicin-induced pain and mechanical allodynia in healthy volunteers with only mild, transient side effects [138]. In a large, triple-blind trial, tezampanel produced a 69% response rate in acute migraine—superior to placebo and comparable to sumatriptan—while remaining well tolerated [139]. Its oral prodrug, NGX426, similarly decreased capsaicin-evoked pain with minimal side effects [140]. A subsequent postoperative dental pain study confirmed that intravenous LY293558 effectively reduces pain—especially evoked pain—while causing only mild, reversible side effects. This trial provided the first clinical evidence that blocking AMPA/kainate receptors can lessen pain and suggested that evoked pain measures may be more sensitive than spontaneous pain ratings for evaluating new analgesics [141].

Overall, pharmacological, genetic, and clinical studies collectively underscore the significant contribution of KARs to the modulation of pain signaling pathways.

## Concluding Remarks

Kainate receptors are increasingly recognized as modulators of nociceptive processing, acting through both pre- and postsynaptic mechanisms in primary sensory neurons and spinal circuits. Recent transcriptomic datasets, however, revealed a complex and subtype-specific expression pattern of KAR subunits, underlining their potential roles beyond classical excitatory transmission. Despite substantial progress, many questions remain regarding how individual subunits may shape pain signaling, how their function changes in pathological states, and whether they can be selectively targeted without disrupting physiological sensory processing. Another important question is whether KAR antagonists or agonists might be effective in modulating pain. So far, only antagonists have been tested. However, given the strategic localization of certain KAR subunits in regulating glutamate or GABA release within pain-related circuits, it is possible that either approach—antagonism or agonism—could ultimately prove more effective as an analgesic. Experimentally addressing these gaps will be essential to determine whether KARs are significant players in sensory transmission and/or represent viable therapeutic targets for the treatment of chronic pain.
